# Identification of CD146 as a marker enriched for tumor-propagating capacity reveals targetable pathways in primary human sarcoma

**DOI:** 10.18632/oncotarget.5375

**Published:** 2015-10-26

**Authors:** Qingxia Wei, Yuning J. Tang, Veronique Voisin, Shingo Sato, Makoto Hirata, Heather Whetstone, Ilkyu Han, Laurie Ailles, Gary D. Bader, Jay Wunder, Benjamin A. Alman

**Affiliations:** ^1^ Department of Orthopaedic Surgery, Duke University, Durham, NC, USA; ^2^ Program in Developmental and Stem Cell Biology, Hospital for Sick Children, Toronto, ON, Canada; ^3^ The Donnelly Centre, University of Toronto, Toronto, ON, Canada; ^4^ Princess Margaret Cancer Centre, University Health Network, Toronto, ON, Canada; ^5^ Department of Medical Biophysics, University of Toronto, Toronto, ON, Canada; ^6^ Department of Molecular Genetics, University of Toronto, Toronto, ON, Canada; ^7^ Samuel Lunenfeld Research Institute, Division of Orthopaedic Surgery, Department of Surgery, Mount Sinai Hospital, Toronto, ON, Canada; ^8^ University Musculoskeletal Oncology Unit, Division of Orthopaedic Surgery, Department of Surgery, Mount Sinai Hospital, Toronto, ON, Canada; ^9^ Department of Laboratory Medicine and Pathobiology, University of Toronto, Toronto, ON, Canada

**Keywords:** human sarcoma, tumor propagating cells, CD146, cell signaling, self-renewal

## Abstract

Tumor-propagating cells (TPCs) are believed to drive cancer initiation, progression and recurrence. These cells are characterized by enhanced tumorigenicity and self-renewal. The ability to identify such cells in primary human sarcomas relies on the dye exclusion ability of tumor side population (SP) cells. Here, we performed a high-throughput cell surface antigen screen and found that CD146 is enriched in the SP population. *In vivo* serial transplantation assays showed that CD146^+^ cells are highly tumorigenic, capable of self-renewal and thus enriches for the TPC population. In addition, depletion of SP cells from the CD146^+^ population show that CD146^+^ cells and SP cells are a distinct and overlapping TPC populations. Gene expression profiling of CD146^+^ and SP cells revealed multiple pathways commonly upregulated in both of these populations. Inhibition of one of these upregulated pathways, Notch signaling, significantly reduced tumor growth and self-renewal. Our data demonstrate that CD146 is an effective cell surface marker for enriching TPCs in primary human sarcomas. Targeting differentially activated pathways in TPCs may provide new therapeutic strategies for treating sarcoma.

## INTRODUCTION

Sarcomas are a diverse group of tumors that arise in the mesenchymal tissues. Osteosarcoma is the most common primary bone sarcoma and undifferentiated pleomorphic sarcoma (UPS), previously known as malignant fibrous histocytoma, is the most common adult soft tissue sarcoma. Similar to many human cancers, osteosarcoma and UPS both display substantial intratumoral heterogeneity. Previous studies found that sarcomas contain a small subpopulation of cells known as tumor-propagating cells (TPCs), characterized by enhanced tumorigenicity and self-renewal capacity [1–3]. TPCs have been hypothesized to drive tumor initiation and progression [4, 5]. Therefore, selective targeting of these cells may be an effective treatment strategy.

The identification of TPCs in many tumor types relies on flow cytometry analysis of specific stem cell markers. For sarcomas, the lack of clearly defined mesenchymal stem cell markers has limited the identification of TPCs. Previous studies used markers that were known to enrich for TPCs in other cancers, such as CD133 and ALDH [6, 7]. However, these studies were performed using cell lines, and did not consistently demonstrate robust *in vivo* serial transplantation capacity [3, 7, 8]. Another approach is to use functional properties to enrich for sarcoma TPCs, such as the side population (SP) assay [2, 9]. This assay is based on the ability of stem-like and progenitor cells to efflux Hoechst dye. Cells that can exclude the dye from their nucleus are termed SP cells, and have been shown to have both increased tumorigenicity and self-renewal ability compared to self-renewal ability compared to non-side population (NSP) cells that make up the bulk of the tumor. However, dye efflux is a dynamic process, and the lack of specific criteria and guidelines for delineating the SP fraction can lead to large variability between studies [10]. As such, a cell surface marker would be of important utility for sarcoma TPC research.

Self-renewal is a defining characteristic of TPCs and is associated with tumor recurrence [4, 11]. Expressions of genes that regulate self-renewal of normal stem cells are significant predictors of disease relapse [12–14]. Currently, the clinical outcome of patients with recurrent or metastatic sarcoma remains poor [15]. The inhibition of self-renewal in sarcoma TPCs may offer valuable targets of therapy.

Here, we used a flow cytometry screen to identify cell surface markers enriched on SP cells compared to bulk tumor cells. We found CD146 (also known as MCAM or MUC18), can reliability enrich for TPCs in osteosarcoma and UPS. Importantly, we showed that CD146^+^ and SP cells are independently tumorigenic and represent overlapping and distinct populations of sarcoma TPCs. Furthermore, pathway analysis revealed that Notch signaling is activated in both of these two TPC populations in osteosarcoma. Treatment with a γ-secretase inhibitor significantly reduced the tumor growth and self-renewal capacity of human osteosarcoma *in vivo*.

## RESULTS

### Flow cytometry screen of cell surface antigens in SP cells

SP cells are significantly enriched for TPCs in sarcomas [1, 2, 9, 16, 17]. To identify cell surface marker(s) that might enrich for TPCs, we performed a high throughput flow cytometry screen of cell surface proteins on the SP cells. Two primary human UPS samples and 1 primary bone sarcoma were obtained from patient biopsy and processed into single cell suspensions. The cells were stained with Hoechst33342 dye to sort for the SP cells. We screened 235 cell surface antibodies, and found five markers that were enriched by greater than 4-fold in the SP population compared to the NSP cells. Specifically, 25.13% (±13.64%SEM) of the SP population expressed CD31, 29.51% (±15.01% SEM) expressed CD66, 11.02% (±3.46% SEM) expressed CD104, 36.34% (±24.27% SEM) expressed CD144 and 16.60% expressed CD146 (±8.10% SEM). In the NSP population, 1.37% (±0.52 SEM) expressed CD31, 0.75% (±0.24% SEM) expressed CD66, 2.47% (± 1.63% SEM) expressed CD104, 0.95% (±0.62%) expressed CD144, and 4.62% (±1.47%) expressed CD146.

### An antibody to CD146 identified a population of sarcoma cells enriched in SP cells

We tested the ability of each of the 5 markers to enrich for tumorigenic sarcoma cells by transplanting the cells *in vivo*. The marker-positive cells were FACS sorted from a UPS and an osteosarcoma sample, and subcutaneously injected at dilutions ranging from 100 to 10,000 cells into NOD-*scid IL2rγnull* (NSG) mice. After 20 weeks, the mice were sacrificed, and the tumors that formed were weighed and examined by histologic examination. CD31^+^, CD66^+^, CD104^+^ and CD144^+^ cells did not show higher tumor initiating ability compared to their respective marker negative populations or bulk tumor cells (data not show). In contrast, CD146^+^ cells enriched for TPCs close to 50-folds higher than CD146^−^ cells.

**Figure 1:**
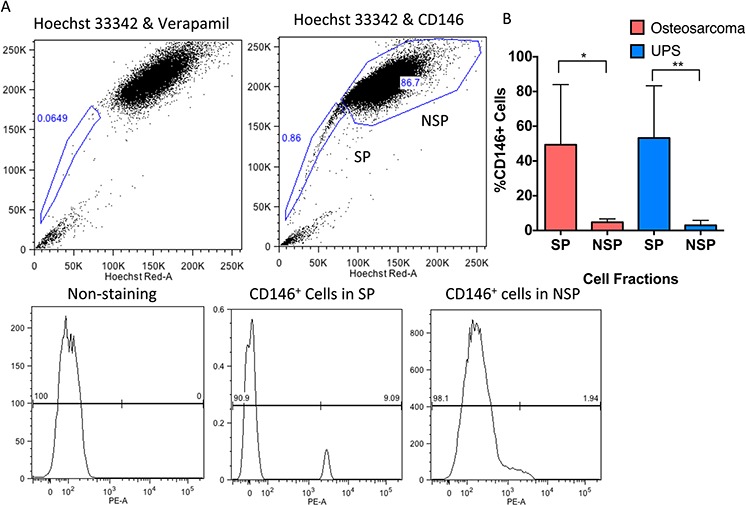
CD146 expression is enriched on the surface of SP cells in human UPS and osteosarcoma **A.** Representative flow cytometry analysis of SP, NSP, and enrichment of CD146 on SP cells in human sarcoma. The NSP is labeled with a box in the upper right quadrant, and SP is in the lower left quadrant. Treating the cells with verapamil inhibits Hoechst dye exclusion, and is used as a negative control for SP analysis. Expression of CD146 is gated on the SP and NSP cells. **B.** Analysis of CD146 expression on SP and NSP cells in 10 primary human UPS samples and 5 primary human osteosarcoma samples, showing CD146 is significantly enriched on the sarcoma SP cells. *P < 0.05; *P < 0.01.

We then analyzed the expression of CD146 using flow cytometry in an independent cohort of 10 human UPS samples and 5 human osteosarcoma samples. The mean percentage of SP and CD146 cells in UPS is 0.70% (±0.16%SEM) and 3.63%(±0.95%SEM) respectively, per tumor. The expression of CD146 was significantly enriched in the SP population compared to the NSP cells (*P* < 0.001), with 53.2% (±9.51% SEM) of SP cells expressing CD146, and 2.98% (±0.90% SEM) of NSP cells expressing CD146 (Figure 1A, 1B). We observed 1 UPS sample (UPS106) with higher percentage of CD146^+^ cells in the NSP populations than the SP population (Supplementary Table S1). This was likely due to the heterogeneity among different patient tumor biopsies. In osteosarcoma, the mean percentage SP and CD146^+^ cells is 0.68% (± 0.28 SEM) and 4.92% (±0.90 SEM) respectively. Similar to UPS, 49.37% (±15.48% SEM) of SP cells express CD146, as compared to 4.73% (±0.87% SEM) of NSP (*P* < 0.05, Figure 1B, Supplementary Table S2). Overall, the enrichment of CD146^+^ cells in SP suggests that there is an overlapping population of CD146^+^ cells and SP cells.

The location of CD146^+^ cells in UPS and osteosarcoma was visualized using immunofluorescence. Since CD146 is also a marker of pericytes [18], we stained frozen primary patient tumor sections with CD31 and CD146 to distinguish between vascular CD146^+^ cells and tumor cells. We found that CD146^+^ cells were present both near blood vessels, and in the tumors away from the vasculature that were CD31^−^, consistent with the presence of a population of tumor cells CD146 (Figure S1).

### CD146^+^ cells show increased tumorigenicity

The ability of CD146^+^ cells to initiate tumors was tested in 5 additional primary human UPS and 5 primary osteosarcoma samples using limiting dilution xenograft assay. As few as 10 CD146^+^ cells in UPS formed tumors at high frequency. In contrast, only a few mice developed tumors when injected with 1, 000 UPS cells from the CD146^−^ fraction (Table 1). Similarly, the CD146^+^ cells in osteosarcoma exhibited substantially higher tumor-forming capacity compared to the CD146^−^ cells (Table 2).

**Table 1:** Serial transplantation of CD146^+^ cells in primary human UPS tumors

Cell type	Cell number	Number of samples tested	Primary mice with tumors/total mice number injected	Secondary mice with tumors/total mice number injected	Tertiary mice with tumors/total mice number injected	Total number of mice with tumors (%)
CD146^+^	1 × 10	5	4/19	2/12	1/8	7/39 (17.9)
	1 × 10^2^	5	8/30	3/12	2/12	13/54 (24.1)
	1 × 10^3^	5	13/24	7/10	3/12	23/46 (50.0)
	1 × 10^4^	5	14/25	10/10	8/8	32/43 (74.4)
CD146^−^	1 × 10	5	0/22	0/14	0/8	0/34 (0)
	1 × 10^2^	5	0/30	0/12	0/12	0/54 (0)
	1 × 10^3^	5	2/24	3/10	0/12	5/46 (10.9)
	1 × 10^4^	5	13/26	7/10	6/8	26/44 (59.1)

**Table 2:** Serial transplantation of CD146^+^ cells in primary human osteosarcoma tumors

Cell type	Cell number	Number of samples tested	Primary mice with tumors/total mice number injected	Secondary mice with tumors/total mice number injected	Tertiary mice with tumors/total mice number injected	Total number of mice with tumors (%)
CD146^+^	1 ×10	5	2/30	2/40	1/12	7/39 (17.9)
	1 × 10^2^	5	6/28	10/46	2/12	13/54 (24.1)
	1 × 10^3^	5	13/30	21/46	3/10	23/46 (50.0)
	1 × 10^4^	5	18/22	20/26	8/10	32/43 (74.4)
CD146^−^	1 × 10	5	0/30	0/44	0/12	0/86 (0)
	1 × 10^2^	5	0/30	0/52	0/12	0/94 (0)
	1 × 10^3^	5	1/28	1/43	0/10	2/81 (2.5)
	1 × 10^4^	5	15/18	8/37	6/10	29/65 (44.6)

### CD146^+^ cells are capable of self-renewal and generate tumors that recapitulate parent tumor

**Figure 2:**
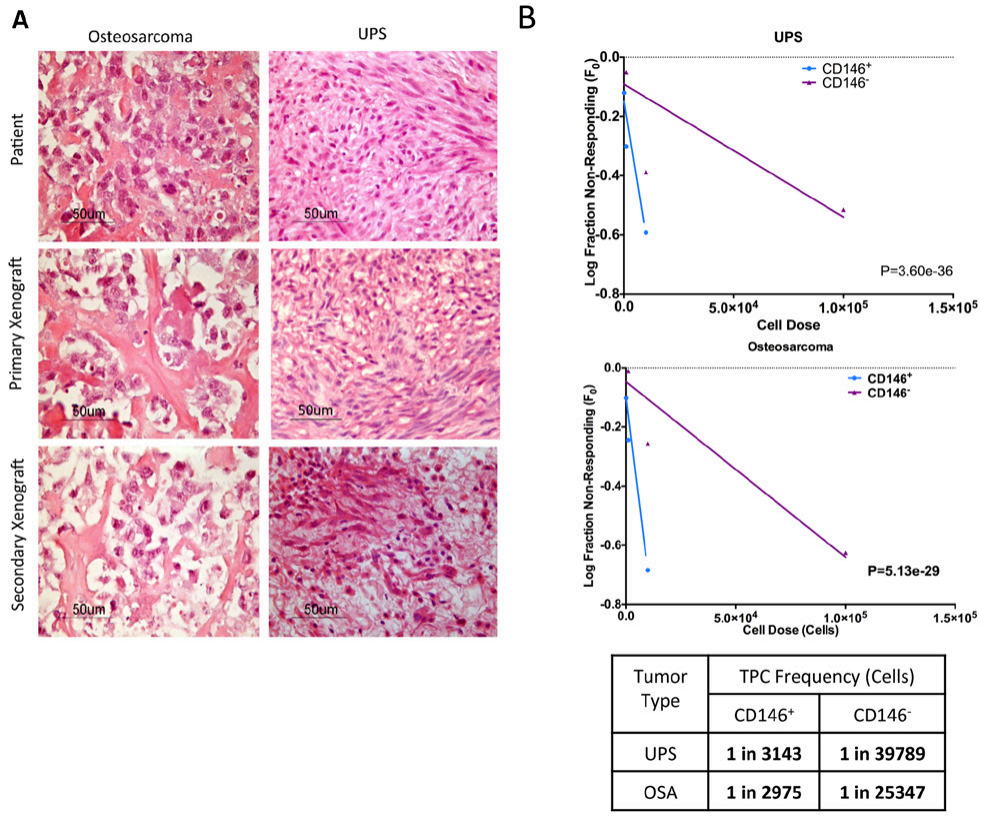
CD146 enriches for sarcoma TPCs **A.** Original patient sarcoma samples obtained from biopsy, primary and secondary xenograft tumors derived from the CD146^+^ cells are formalin-fixed, paraffin-embedded and stained with hematoxylin and eiosin (H&E). The grafted tumors are identical histologically compared to the original patient tumor. **B.** Graphical representation of *in vivo* limiting dilution assay, which compares TPC frequency in CD146^+^ cells and CD146^−^ cells. Tumor initiation data from secondary injection of CD146^+^ and CD146^−^ cells into NSG mice are used to determine the enrichment of TPCs in each population. CD146^+^ cells significantly enriches for TPCs in UPS and osteosarcoma compared to CD146^+^ cells.

A fundamental characteristic of TPCs is the ability to self-renew and to recapitulate the histological characteristics of the parent tumor [19]. As such, we serially injected CD146^+^ and CD146^−^ cells from primary xenografts into fresh NSG mice. In secondary and tertiary transplants, CD146^+^ cells continued to exhibit enriched tumor-forming ability compared to CD146^−^ cells (Tables 1 and 2). Furthermore, hematoxylin and eosin staining of tumors xenograft derived from CD146^+^ cells resembled the patient tumor in both UPS and osteosarcoma (Figure 2A). To determine the frequency of TPC enrichment in CD146^+^ fraction, we used the Extreme Limiting Dilution Analysis (ELDA) algorithm on data from the secondary transplants [20]. For UPS, the frequency of TPCs in the CD146^+^ fraction is 1/555 cells, compared to 1/17002 cells in CD146^−^ fraction, indicating a 30.6-fold enrichment (*P* = 3.60e-36, Figure 2B). For osteosarcoma, the TPC frequency is 1/2830 cells and 1/68375 cells in CD146^−^ fraction, indicating a 24.2-fold enrichment (*P* = 5.13e-29, Figure 2B).

Taken together, these data show that CD146 reliably identifies a population of cells in sarcoma that are enriched for TPCs, which are characterized by enhanced tumorigenicity, the ability to self-renewal, and the ability to initiate tumors that resembles the parent tumor.

### CD146^+^ enriches for a distinct TPC population from SP cells

Since CD146 was identified based on its enrichment in the SP population, we examined whether if the tumorigenic and self-renewal capacity of CD146^+^ cells are dependent on the SP population. We isolated the NSP population following Hoechst dye staining, and sorted for CD146^+^ and CD146^−^ fractions. The sorted cells were injected into NSG mice at different dilutions. We found that the NSP CD146^+^ population was significantly more tumorigenic than the NSP CD146^−^ population over serial transplants (Table 3, *P* = 9.58e-13). Therefore, despite the overlap between SP and CD146^+^ population, CD146^+^ cells depleted of SP remain enriched for tumor propagating capacity, suggesting that CD146 enrich for a subpopulation of TPCs in sarcoma that both overlap with, but also distinct from SP cells.

**Table 3:** Serial transplantation of different sarcoma cell fractions

Cell Type	Cell Number	Primary mice with tumors/total mice number injected	Secondary mice with tumors/total mice number injected	Total number of mice with tumors (%)
NSP CD146^+^	1 × 10	0/12	3/6	3/18 (16.7)
	1 × 10^2^	9/12	6/6	15/18 (83.3)
	1 × 10^3^	7/7	4/4	11/11(100)
	1 × 10^4^	3/3	4/4	7/7 (100)
NSP CD146^−^	1 × 10	0/12	0/6	0/18 (0)
	1 × 10^2^	2/12	1/6	3/18 (16.7)
	1 × 10^3^	6/10	2/4	8/14 (57.1)
	1 × 10^4^	8/8	4/4	12/12 (100)

### Common signaling pathways are upregulated in CD146^+^ and SP populations

**Figure 3:**
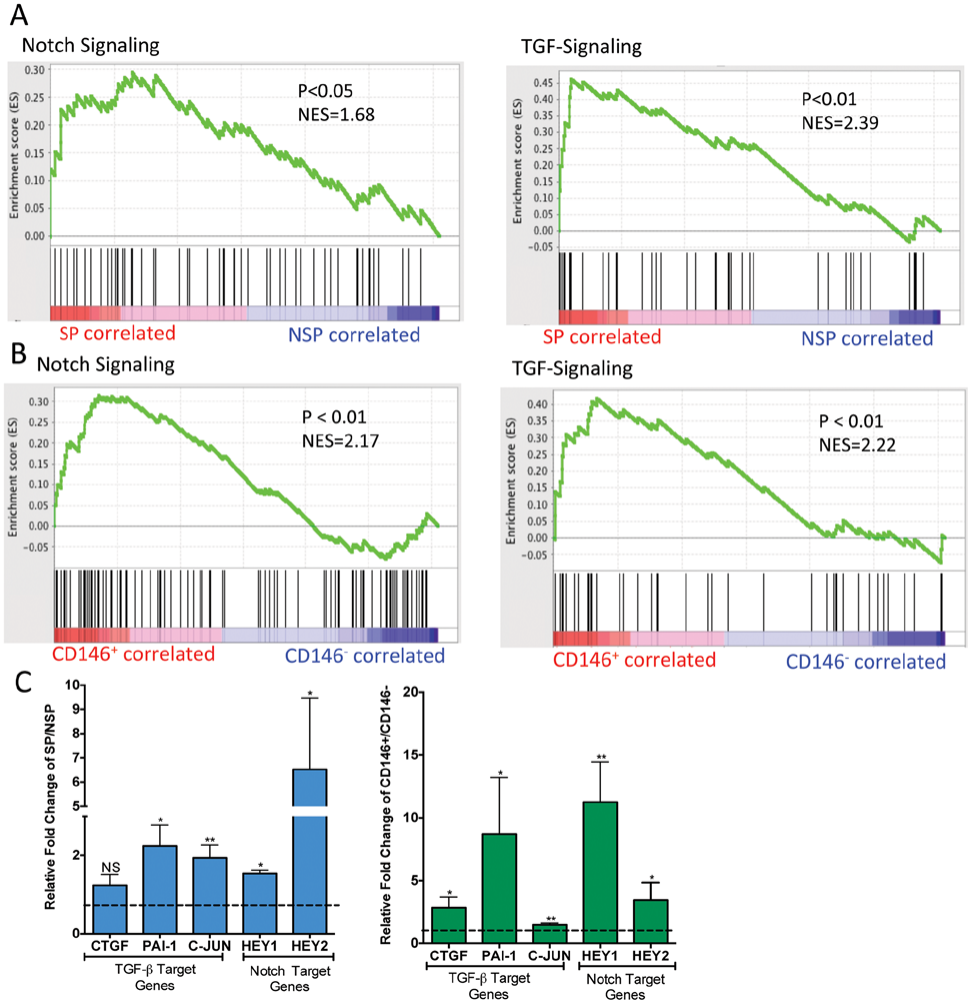
Notch and TGF-β signalling pathways are significantly upregulated in SP and CD146^+^ cells GSEA analysis comparing SP versus NSP cells **A.** and CD146^+^ versus CD146^−^ **B.** cells for enrichment of Notch and TGF-β signalling pathway gene sets. **C.** Quantification of Notch and TGF-β signalling pathway target genes in SP versus NSP cells and CD146^+^ versus CD146^−^ cells by qPCR. *P < 0.05; ** < 0.01.

To further examine similarities and differences between the cell populations, gene expression profiling was undertaken to compare the various cell fractions. This data was used to identify cell-signaling pathways that are differentially regulated in the same manner in both CD146^+^ and SP cells. Four separate populations of CD146^+^, CD146^−^, SP and NSP were sorted from 3 osteosarcoma samples, and their expression profiles were analyzed. Using a 1.5 fold change and a *P* < 0.05 as thresholds, we identified 3763 differentially expressed genes between CD146^+^ versus CD146^−^ cells and 757 differentially expressed genes between SP versus NSP cell. Using Gene Set Enrichment Analyses (GSEA), we performed pathways analysis for CD146^+^ versus CD146^−^ cells and SP versus NSP cells. This analysis identified multiple targetable pathways that were similarly affected in the SP and CD146^+^ populations (Figure S2). In particular, TGF-β and Notch signaling pathways, both known to regulate stem cell properties and contribute to tumorigenesis [1, 21, 22] were significantly enriched in upregulated genes (Figure 3A and 3B). To validate this, the expression of known TGF-β and Notch signaling target genes were measured by qPCR in SP and CD146^+^ fractions relative to their negative counterparts (Figure 3C). Comparison between CD146^+^ and CD146^−^ cells showed significant upregulation of CTGF, c-JUN, PAI-1, HEY1, and HEY2. With the exception of CTGF, these target genes were also significantly upregulated in the SP population [19, 23].

This analysis also identified pathways with different enrichment results between SP and CD146^+^ cells. Specifically, FGFR, camodulin, CREB and phospholipase C associated signaling were enriched in SP cells but not CD146^+^ cells (Figure S2A). On the other hand, extracellular matrix remodeling, cell migration, hypoxia response and angiogenesis associated pathways were enriched only in the CD146^+^ cells (Figure. S2B). These differences are consistent with the CD146^+^ and SP having distinct populations.

### Inhibition of Notch signaling reduces tumor growth and self-renewal

**Figure 4:**
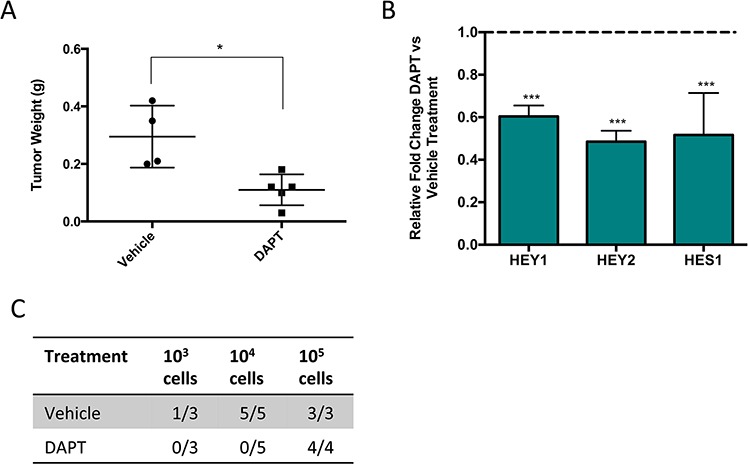
Pharmacological inhibition of Notch signaling significantly reduces osteosarcoma growth and self-renewal **A.** Tumor weight of mice bearing human osteosarcoma following 3 weeks of DAPT compared to vehicle treatment. **B.** Relative gene expression of Notch target genes in DAPT treated mice compared to vehicle treated mice. **C.** Tumors treated with DAPT or vehicle are digested and injected into fresh NSG mice at known concentrations to evaluate the effect of DAPT on self-renewal. The number of secondary tumors is counted after 16 weeks of observation.

The self-renewal capacity of TPCs has been hypothesized to contribute to tumor recurrence [4]. To determine if pathways enriched in both SP and CD146 positive cells would drive self-renewal, we examined one such pathway, Notch signaling using a pharmacological inhibitor. This signaling pathway is known to regulate mesenchymal stromal cells (MSCs) differentiation [1, 24, 25]. NSG mice bearing primary human osteosarcoma cells were treated with DAPT, a γ-secretase inhibitor that targets Notch signaling. After 3 weeks of treatment, mice that received DAPT showed significantly smaller tumor sizes compared to the vehicle treated mice, and the expression of Notch target genes were reduced in the treated tumors (Figure 4A, 4B). Next, we compared the self-renewal potential of these cells by examining the tumor-initiating potential of DAPT and vehicle treated cells in fresh NSG mice. DAPT treated cells showed significantly reduced ability to initiate tumors (Figure 4C). Thus, Notch signaling drives self-renewal in osteosarcoma, and its inhibition, using agents such as DAPT may serve as a potential therapy against sarcoma self-renewal.

## DISCUSSION

In this study, flow cytometry screen of cell surface markers on sarcoma SP cells and functional validation by serial transplantations identified CD146 as a marker enriching for tumor propagating capacity in UPS and osteosarcoma. This is the first cell surface marker identified in primary sarcoma samples. Previously identified cell markers in sarcomas have not been tested in human patient samples using the gold standard *in vivo* serial transplantation assay for evaluating self-renewal. Since CD146 can be readily detected with various approaches using antibodies, it has significant advantages compared to the SP analysis, which is a technically challenging method that often produces inconsistent results from the same sample.

CD146 is a cell-adhesion molecule that belongs to the immunoglobulin superfamily [26]. It was first identified as a specific antigen for human malignant melanoma, and its expression was subsequently shown to associate with poor survival, tumor progression, and metastasis in other cancers [27–29]. Certain MSC populations, such as pericytes, also express CD146 [18]. We identified CD146 based on its expression in the SP cells, a population that has been previously shown by multiple studies to be highly enriched for TPCs [1, 2, 16, 17, 30]. CD146^+^ cells in sarcomas are relatively more abundant representing 3.61% of UPS and 4.91% of osteosarcoma. The ability of CD146^+^ to initiate tumors over multiple transplantations suggests it indeed enriches for TPCs with self-renewal ability. However, in many human cancers where TPCs has been identified, the percentage of these cells is generally less than 0.04% [31]. In theory, a single TPC is sufficient to generate tumors. The reduced ability of CD146^+^ cells to form tumors at the 10 cell dose suggest that even though this population is enriched for TPCs, it may contain other cells such as transit amplifying cells.

Our serial transplantation data showed that CD146^+^ cells possess TPC properties in the absent of SP cells. This indicates that the TPC population in UPS and osteosarcoma may be heterogeneous, and the NSP CD146^+^ cells are a distinct TPC population with some overlap in the SP population. The heterogeneity of TPCs also suggests that these populations may respond differently to targeted therapy. Indeed, Kreso *et al* found that different clonal populations with TPC properties in colorectal cancer vary in their proliferative potential, self-renewal ability and response to therapy [32]. As such, identification of common pathways in the heterogeneous TPC populations may offer one approach to identify effective targets of therapy.

Using gene expression profiling and pathway analysis, we showed that Notch signaling is activated in both SP and CD146^+^ cells. Treatment of patient derived xenograft in mice with DAPT, a known inhibitor of Notch signaling, significantly reduced primary tumor growth. More importantly, DAPT inhibited the tumors from growing back in secondary transplants, even with treatment withdrawal, demonstrating that this treatment targets self-renewal. The importance of Notch signaling in osteosarcoma initiation is further substantiated by recent work from Tao *et al*, which showed that over-expression of Notch-1 Intracellular Domain in MSCs and osteoblast progenitor cells drives osteosarcoma formation in mice [21]. In addition, we found other self-renewal pathways activated in SP and CD146^+^ cells. Hedgehog and YAP signaling pathways may be activated in CD146^+^ cells. TGF-β signaling, which participates in stem-cell maintenance is upregulated in both SP and CD146^+^ cells [22]. Wnt activation is observed in SP cells, but was not statistically significant in our data. This suggests that self-renewal pathways may be heterogeneous in different tumor samples. Furthermore, our pathway analysis showed that metabolic pathways, especially, glucose metabolism are upregulated in the SP and CD146^+^ cells. The increased glucose uptake of TPCs compared to bulk tumor cells may contribute to their survival advantage [33–35]. Future investigations to target signaling pathways differentially regulated in TPCs may have therapeutic value.

The tumor microenviroment plays important roles in regulating the properties of TPCs [36]. Emerging evidence suggest that stromal cells, non-TPC tumor cells, and the extracellular matrix provide important signaling molecules that supports TPCs growth, self-renewal, and protects TPCs from immunosurveillance [5, 37]. A potential limitation of the xenograft model is that certain aspects of the tumor microenvironment may not be fully recapitulated in the recipient animal. This may select for certain cells that are more suited for the transplant environment, affecting the accurate estimation of cells with tumor propagating capacity [38]. Developments of more immunodeficient mice, and humanized mice that expresses signaling molecules found in the tumor microenvironment may allow more accurate assessment of TPCs. Alternatively, lineage tracing of TPCs in transgenic mouse models of sarcomas may allow us to further characterize the role of TPCs in their native environment.

Our study identified CD146 as a cell surface marker for cells enriched in TPC properties in primary human sarcomas. Using *in vivo* model, we demonstrated that CD146^+^ tumor cells show increased tumorigenicity, self-renewal ability, and can initiate tumors that resemble the primary patient tumor. Furthermore, the tumorigenic potential of CD146^+^ cells is also independent of the SP fraction, a known TPC enriched population, suggesting that the sarcoma TPC population may be heterogeneous. Our gene expression profiling and pathway analysis of CD146^+^ and SP cells identified Notch signaling is a potential target for inhibiting osteosarcoma self-renewal. Given the potential heterogeneity of TPCs, in addition to CD146, other novel markers of TPCs may exist. Future identification and characterization of sarcoma TPCs may reveal new targets of therapy.

## MATERIALS AND METHODS

### Primary tumor samples

Human undifferentiated pleomorphic sarcoma (UPS) and osteosarcoma tumor tissues were obtained at the time of initial biopsy, prior to any therapy. A musculoskeletal pathologist verified the diagnosis of the tumors. To generate single-cell suspensions, the samples were mechanically dissociated into small pieces, and enzymatically digested with a mixture of 10mg/mL of collagenase IV (Worthington), 2.4U/ml of Dispase (Becton Dickinson), and 0.05% trypsin (Wisent) for 45–60minutes at 37°C, followed by filtering through a 70 μm strainer as previously reported [1, 2]. Hematopoietic cells were excluded with whole blood lysis buffer (Life Technologies). Afterwards, the single cell suspensions were stained with anti-human CD45-PE-Cy7 antibody (1:200, Becton Dickinson) to deplete the immune cells via flow cytometry activated cell sorting (FACS).

### Flow cytometry

Side population (SP) cells were collected as previously described [2]. Briefly, single-cell suspensions were treated with 2.5 mg/mL of Hoechst 33342 dye (Sigma) alone, or in combination with 50 mmol/L of verapamil (Sigma) as a negative control, for 90 minutes at 37°C. SP, cells were identified using dual wavelength analysis (blue, 424–444 nm; red, 675 nm) after excitation with 350 nm UV light (MoFlowXDP). Staining with CD45 antibody (BD Pharmingen) was used to eliminate hematopoetic cells. To sort for CD146^+^ cells, processed tumor cells were stained with anti human CD146-PE conjugated antibody at 1:100 dilution for 30 minutes at 4°C (BD Pharmingen). Murine cells, which makes up the tumor stroma in the xenografts were excluded from staining with a biotin conjugated anti-mouse H-2k^d^ antibody (BD Pharmingen) streptavidin PE-Cy7 conjugate (Invitrogen) at 1:1,000 for 30 minutes at 4°C. H-2k^d+^ cells were removed from analysis during FACS. Since the stromal cells are likely derived from the mouse, removing the H-2k^+^ cells allow us to deplete the stromal cells from the tumor [1, 2]. In all flow cytometry experiments, cells were counterstained with 1 mg/mL of propidium iodide (PI; Molecular Probes) and the dead cells were removed from the analysis.

### Flow-cytometry cell surface antigen screen

The SP and NSP suspensions were incubated on ice in flow cytometry buffer (FC buffer: Hanks balanced salt solution with 1% BSA, 2 mM EDTA). The cells were aliquoted into round-bottom 96-well plates containing 235 fluorochrome-conjugated cell-surface targeted antibodies (*i.e*. each of 235 wells contained a different antibody, Supplementary Table S3). Antibodies were labeled with PE, FITC or APC. Wells containing buffer only were included as negative controls. Cells were suspended at a concentration to achieve a minimum cell number of 50,000 cells per well in a final volume of 200 ul per well. The final antibody dilution was 1:50 for all antibodies. Once cells were added to the wells, plates were incubated on ice in the dark for 20 minutes. Plates were centrifuged to pellet cells, buffer aspirated, and pellets were washed twice with 200 ul of FC buffer. Finally, pellets were suspended in 100 ul of FC buffer containing 1 ug/ml of propidium iodide to allow exclusion of dead cells. Cells were then analyzed on a BD LSRII equipped with a high throughput sampler. A minimum of 10,000 events per well were collected on FACSDiva software, and resulting FCS 3.0 files exported to FlowJo version 9.3 for analysis. Dead cells, debris, cell doublets and CD45^+^ immune cells were excluded from the analysis, after which side-population and bulk tumor cells were analyzed for expression of each of the 235 markers. Enrichment of a cell surface marker was determined by comparing the percent of marker- positive cells in the SP population to the percent of marker-positive cells in bulk tumor cells. Markers with an enrichment of ≥ 4-fold in two of the three samples analyzed were selected for testing *in vivo*.

### Limiting dilution assay

Individual cells sorted from flow cytometry according to various markers including CD146 were suspended in PBS. Murine cells from xenografts were excluded during FACS by staining with a biotin-conjugated anti-mouse H-2k antibody (BD Pharmingen). The sorted cell counts from flow cytometry were manually confirmed with a hemocytometer and then serially diluted in PBS to achieve cell range between 10–10^5^ cells/100 μl. For each injection, the cells were mixed with an equal volume of ice-cold Matrigel (Becton Dickinson) and subcutaneously injected into 6- to 8- week-old NOD-*scid IL2rγnull* (NSG) mice. After injection, the mice were observed for 24 weeks and the tumors were dissected. The tumors were weighed using an analytical balance and were examined histologically. Tumor-propagating cell frequency was calculated based on extreme limiting dilution data from the secondary tumor transplantations as described in [20]. The model follows standard general linearized models to compare the frequency of TPCs in one or more populations, and allows for one-sided confidence intervals if 0% or 100% positive responses are observed [20, 39]. The model can be accessed online at http://bioinf.wehi.edu.au/software/elda/ from Walter and Eliza-Hall Institute [20].

### Histology and immunofloresence

Tumor biopsies from patients and mice xenografts were formalin fixed and paraffin embedded. Tumor sections were cut at 8 μm and stained with hematoxylin and eosin following standard procedure and observed in a blinded manner. At least 5 sections from each tumor sample were analyzed. For immunofloresence, fresh tumors biopsies from patients were embedded in Tissue Tek O.C.T compound (Fisher Scientific) and snap frozen in dry ice. Sections of 8 μm were cut and blocked with 10% donkey serum, 2%BSA, in 1X PBST for 1 hour at room temperature. Tissue slides were incubated with CD31 antibody (Abcam) at 1:50 dilution for 1 hour. This was followed by secondary antibody labeling with Alexa488 goat anti-rabbit (Life Technologies) at 1:1000 dilutions for 30 minutes. CD146 (BD Pharmagen) were diluted at 1: 5000 and incubated on the tissue slides for 30 minutes, followed by Alexa594 donkey anti-mouse antibody at 1:1000 dilutions for 30 minutes. The slides were mounted with mounting media containing DAPI (Vectashield, Vector Laboratories Inc) and imaged.

### Gene expression profiling analyses

Three independent human osteosarcoma xenografts were sorted for SP, NSP, CD146^+^ and CD146^−^ fractions. The stromal cells were removed by staining with anti-mouse H-2k antibody (BD Pharmingen). RNA samples were extracted and converted to cDNA using Ovation RNA Amplification System V2 (Nugen) following manufacture's protocol. The cDNA was analyzed using Illumina HT-12 v4 platform following standard protocol. Results were analyzed using *R* (version 3.2.0] with the LIMMA (linear models for microarray data) package [40, 41]. We examined the raw data with a Normexp by Control (neqc) algorithm pre-processing strategy described by Shi et al using *R* [42]. This approach includes background correction, quantile normalization and log2 transformation to the raw data [42, 43]. Differentially expressed genes were identified using LIMMA as described in [44]. Differentially expressed genes were by fitting a linear model for each gene in the data, and then an empirical Bayes (EB) method is used to moderate the standard errors for each gene expression. Genes with a fold-change > 1.5 and *P* < 0.05 were considered significant. The microarray data was submitted to the NCBI GEO database with the identifier GSE63390.

### Gene set enrichment analysis (GSEA)

Pathway analyses were performed using Gene Set Enrichment Analysis (GSEA) with parameters set to 2,000 gene set permutations and gene sets size between 8 and 500 [45]. Gene sets were obtained from KEGG, MsigDB-c2, NCI, Biocarta, IOB, Netpath, Human Cyc, Reactome and the Gene Ontology (GO) databases (http://www.baderlab.org/GeneSets, 46, 47). An enrichment map was generated using Cytoscape with parameters set for a nominal *P* value of < 0.005, FDR < 0.25, and the Jaccard coefficient equal to 0.5 [48].

### Quantitative real-time reverse transcription PCR (qPCR)

Analysis of gene expression using qPCR was performed as previously described [49]. The reactions were performed on ViiA Real-Time PCR System (Applied Biosystems) with Taqman Fast Universal PCR Master Mix (Life Technologies). The primer sets for all the genes was purchased from Life Technologies. The expression level of the genes was calculated using standard ΔΔCt method, with GAPDH as internal control. All reactions were performed with at least 3 replicates.

### *In vivo* DAPT treatment

Eight to 10 weeks old NSG mice were subcutaneously injected on the left flank at 200,000 cells dissolved in Matrigel. After 6 weeks the animals were treated with N-[(3,5-Difluorophenyl)acetyl]-L-alanyl-2-phenyl]glycine-1,1-dimethylethyl ester (DAPT) dissolved in corn oil with 5% DMSO 100 mg/kg/day (Selleck Chemicals, Texas) or vehicle via intraperitoneal injection for 22 days. The mice were euthanized and tumors were harvested. Each tumor was dissected free from surrounding tissues and weighed. The tumors were then digested into single cell suspension, and injected into fresh NSG mice at limiting dilutions, as described above. Secondary transplants were observed for up to 16 weeks.

### Statistical analyses

Statically analyses were performed using GraphPad Prism v6. All results are representative of at least *n* = 3. The data were shown as mean ± standard error of mean (SEM), unless otherwise stated, and was calculated using Prism. Statistical significance was calculated using two-tailed, unpaired Student's *t*-test. A *P* value < 0.05 is considered significant.

## SUPPLEMENTARY FIGURES AND TABLES


