# Supplementary material for: Identification of CD146 as a marker enriched for tumor-propagating capacity reveals targetable pathways in primary human sarcoma
Source: Oncotarget. 2015 Oct 26;6(37):40283–94. doi: 10.18632/oncotarget.5375 (PMC4741895; doi:10.18632/oncotarget.5375)
Supplement: Supplementary file 1 [file oncotarget-06-40283-s001.pdf]

SUPPLEMENTARY FIGURES AND TABLES

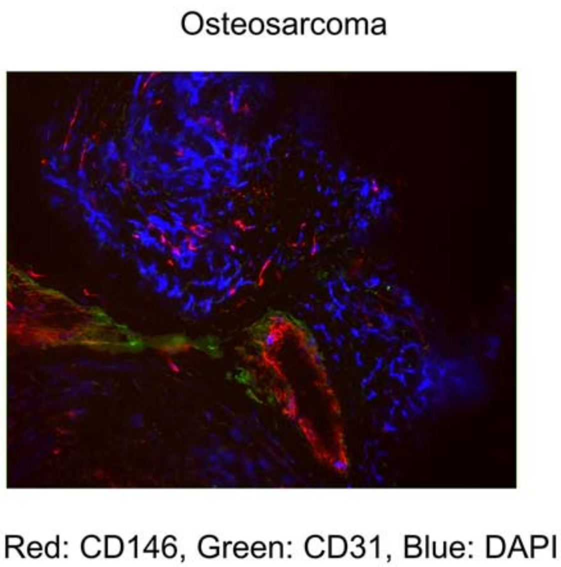

Supplementary Figure S1: Localization of CD146 expressing cells in human Ups and osteosarcoma. Fresh frozen patient biopsies from osteosarcoma and Ups are sectioned and stained with Cd146 (Red), CD31 (Green) and DAPI (Blue).

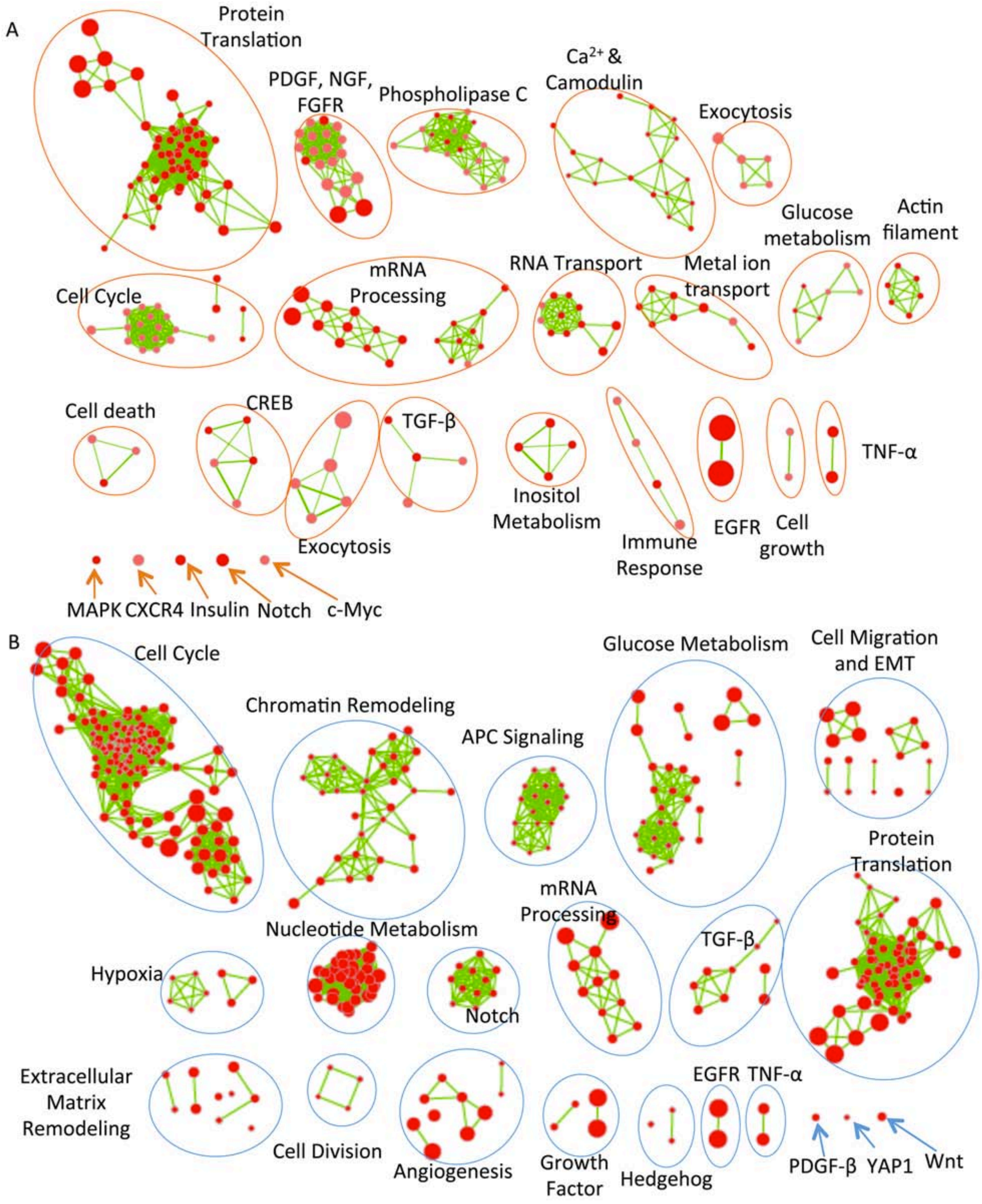

Supplementary Figure S2: Pathway analysis of sp versus NSP cells and CD146+versus Cd146- cells. GSEA results visualized by cytoscape showing pathways enriched in **A.** sp versus NSP cells and **B.** CD146^{+} versus CD146^{-}cells. Nodes represent significantly enriched pathways and green lines connect overlapping pathways.

Supplementary Table S1: Abundance of CD146 positive cells in UPS

	UPS			
Sample ID	Total SP (%)	Total CD146^{+} (%)	CD146^{+} in SP (%)	CD146^{+} in NSP (%)
UPS100	0.54	0.64	55.6	0.34
UPS101	0.90	0.57	33.3	0.27
UPS102	1.41	1.41	49.6	0.72
UPS103	1.34	8.32	74.6	7.42
UPS104	0.01	0.58	80.0	0.50
UPS105	0.05	4.40	65.2	4.37
UPS106	1.13	1.53	0.61	1.55
UPS107	0.66	7.03	75.8	6.57
UPS108	0.14	6.22	85.7	6.11
UPS109	0.86	5.64	9.09	1.94

Supplementary Table S2: Abundance of CD146 positive cells in osteosarcoma

Osteosarcoma				
**Sample ID**	**Total SP (%)**	**Total CD146$^+$ (%)**	**CD146$^+$ in SP (%)**	**CD146$^+$ in NSP (%)**
OS100	0.38	3.86	78.2	3.56
OS101	0.28	6.13	64.2	5.97
OS102	0.40	7.86	80.0	7.57
OS103	1.80	3.55	7.74	3.45
OS104	0.54	3.16	16.7	3.11

Supplementary Table S3: List of cell surface antibodies used for high throughput flow cytometry (HT-FC) Screen

	1	2	3	4	5	6	7	8	9	10	11	12
A	CD1A	CD8	CD15	CD23	CD31	CD41A	CD48	CD53	CD62L	CD70	CD85J	CD97
B	CD1D	CD9	CD16	CD24	CD32	CD42A	CD49A	CD54	CD62P	CD71	CD86	CD98
C	CD2	CD10	CD16B	CD25	CD33	CD42B	CD49B	CD55	CD63	CD73	CD87	CD99
D	CD3	CD11A	CD18	CD26	CD34	CD43	CD49C	CD56	CD64	CD79A	CD88	CD102
E	CD4	CD11B	CD19	CD27	CD35	CD45RA	CD49D	CD58	CD66	CD79B	CD89	CD103
F	CD5	CD11C	CD20	CD28	CD36	CD45RB	CD49E	CD59	CD66C	CD80	CD90	CD104
G	CD6	CD13	CD21	CD29	CD38	CD45RO	CD49F	CD61	CD68	CD81	CD91	CD106
H	CD7	CD14	CD22	CD30	CD40	CD47	CD51	CD62E	CD69	CD83	CD94	CD107A
I	CD108	CD120B	CD132	CD142	CD158A	CD172B	CD196	CSW210	OX40LIG	CD274	CD328	TLR9
J	CD109	CD122	CD134	CD144	CD158B	CD180	CD197	CD212	CD253	B7-H2	NKP46	EGF-R
K	CD110	CD123	CD135	CD146	NKB1	CD181	CD200	CD221	TWEAK	CD278	NKP44	FMLP-R
L	CD112	CD124	CD137	CD150	CD161	CD182	CD201	CD226	CD267	CD279	CD337	FOXP3
M	CD114	CD125	CD138	CD151	CD162	CD183	CD205	CD235A	BAFF-R	LAIR-1	ITGB7	IL21-R
N	CD116	CD126	CD140A	CD152	CD163	CD184	CD206	P-GP	CD271	CD314	SSEA-1	BLTR-1
O	CD117	CD127	CD140B	CD154	CD164	CD193	CD208	CD244	BTLA	F11 RCPT	SSEA-3	ALP BTA T
P	CD119	CD130	CD141	CD157	CD166	CD195	CD209	NPM-ALK	CD273	EPCAM	SSEA-4	GMA DTA
Q	TNFR RLP	HLA-DM	CD1B	CD72	CD227	CD39	HLA-DR	KIR-NKAT2	CD46	CDW93	CLIP	DCIR
R	V BTA8	BTA2-MGL	CD37	CD74	CD282	CD192	HMPT PRGN	CD50	CD107B	FMC7	CD133	
S	V DTA2	MIC A&B	CD41B	CD75	E-CADH	CCR9	HLA-A2	CD57	CD147	CLA		
T	GALECTIN	HER2/ NEU	CD44	CD77	CDW329	CD294	HLA-ABC	CD66B	CXCR5	PAC-1		
